# Highly Sensitive Nuclease Assays Based on Chemically Modified DNA or RNA

**DOI:** 10.3390/s140712437

**Published:** 2014-07-11

**Authors:** Shinobu Sato, Shigeori Takenaka

**Affiliations:** Department of Applied Chemistry and Research Center for Bio-Microsensing Technology, Kyushu Institute of Technology, Kitakyushu, Fukuoka 804-8550, Japan; E-Mail: shinobu@che.kyutech.ac.jp

**Keywords:** nuclease, DNase I, RNase A, oligonucleotide, disease marker, electrochemistry, fluorescence resonance energy transfer (FRET), nanocluster

## Abstract

Nucleolytic enzymes are associated with various diseases, and several methods have been developed for their detection. DNase expression is modulated in such diseases as acute myocardial infarction, transient myocardial ischemia, oral cancer, stomach cancer, and malignant lymphoma, and DNase I is used in cystic fibroma therapy. RNase is used to treat mesothelial cancer because of its antiproliferative, cytotoxic, and antineoplastic activities. Angiogenin, an angiogenic factor, is a member of the RNase A family. Angiogenin inhibitors are being developed as anticancer drugs. In this review, we describe fluorometric and electrochemical techniques for detecting DNase and RNase in disease. Oligonucleotides having fluorescence resonance energy transfer (FRET)-causing chromophores are non-fluorescent by themselves, yet become fluorescent upon cleavage by DNase or RNase. These oligonucleotides serve as a powerful tool to detect activities of these enzymes and provide a basis for drug discovery. In electrochemical techniques, ferrocenyl oligonucleotides with or without a ribonucleoside unit are used for the detection of RNase or DNase. This technique has been used to monitor blood or serum samples in several diseases associated with DNase and RNase and is unaffected by interferents in these sample types.

## 1. Introduction

Nucleases cleave or digest the phosphodiester bonds of DNA [1], RNA [2], and/or DNA-RNA hybrids [3]. They prevent replication errors and eliminate fragmentation of RNA or DNA, which may induce programmed cell death [1–3]. Nucleases prevent invasion or infection by viruses carrying single- or double-stranded nucleic acids, and they are expressed in the saliva, sudoriferous glands, and skin [1–3]. These enzymes are called deoxyribonuclease (DNase) or ribonuclease (RNase) for their respective substrates [1,2,4]. In the laboratory, DNase I is required to remove DNA from samples used in mRNA expression assays, whereas RNase A is used to remove RNA from samples used for DNA analysis. DNase and RNase are important for modifying and metabolizing nucleic acid chains and can be used as disease markers [4–13]. We provide a summary review of the detection methods for DNase and RNase as disease markers (Table 1).

## 2. Characteristics of Nucleases

### 2.1. Characteristics of DNase

DNase I is a typical DNase that cleaves the 3′-phosphate from single- or double-stranded DNA in the presence of Mg^2+^ to generate 5′-phosphate and 3′-hydroxy termini [1]. DNase I cleaves single- and double-stranded DNA with some preference for 5′-pyridimidine purine-pyrimidine sequences. In 1957, DNase I was recognized as a useful marker of *Staphylococcus* infection in human and animal skin [4]. *Staphylococcus aureus* is ubiquitous and is associated with food poisoning, pneumonia, and blood poisoning. Detection of *S. aureus* is important, but distinguishing this pathogen from the common nontoxic, coagulase-negative staphylococci is difficult. DNase produced by *S. aureus* may be a useful marker for this pathogen. For detection, sample bacteria are cultured on selective media containing DNA; the presence of *S. aureus* is indicated by the absence of white turbidity upon addition of hydrochloride. *S. aureus* cultured on agar containing DNA and toluidine blue turns the medium burgundy. These behaviors are associated with DNase activity.

DNase can also be used as a marker of disease. For instance, accumulation of DNA or DNA-protein complexes in the blood causes several autoimmune diseases [5,6]. This accumulation on blood vessel walls, glomeruli, or joints leads to glomerular nephritis, arthritis, or anthema [7]. Individuals with these diseases have been found to express lower levels of DNase I than do normal individuals. In contrast, DNase I activity in blood is higher in patients with breast or oral cancer and lower in patients with lymphatic malignancies or stomach cancer [8]. Increased DNase I activity is a marker of acute myocardial infarction and transient myocardial ischemia. Transarterial chemoembolization is an effective cure for liver cancer, but survival rates are low in patients with blood DNase activity below 21% within 24 h after treatment [9]. DNase is used to monitor treatment response and is used to treat cystic fibroma (CF), in which patients accumulate DNA in the blood. DNase I treatment promotes DNA metabolism [10].

### 2.2. Characteristic of RNase

RNase A is a typical RNase (13,686 Da; 124 amino acids) and was the third protein to be defined by X-ray structure analysis [33,34]; it was chemically synthesized by Merrifield [35], who demonstrated its biological and chemical synthesis. RNase A is an endonuclease that cleaves single-stranded RNA at cytosine (C) or uracil (U) residues, producing 3′-phosphate termini. RNase A has antiproliferative, cytotoxic, and tumor-inhibiting activities and the homologous ranpirnase (ONCONASE™) has been developed as an antineoplastic drug for mesothelial and cutaneous cancers [11,12,36]. In contrast, the RNase angiogenin mediates vascularization to repair damaged blood vessels and during cancer development [37]. Since angiogenin inhibitors block vascularization, it is a candidate anti-cancer drug. Thus, RNase A is an important target for drug discovery. RNase is also a candidate disease marker for ovarian tumors and thyroid and pancreatic cancer [4,5].

## 3. Detection of DNase

### 3.1. Fluorometric DNase Detection

**Figure 1.**
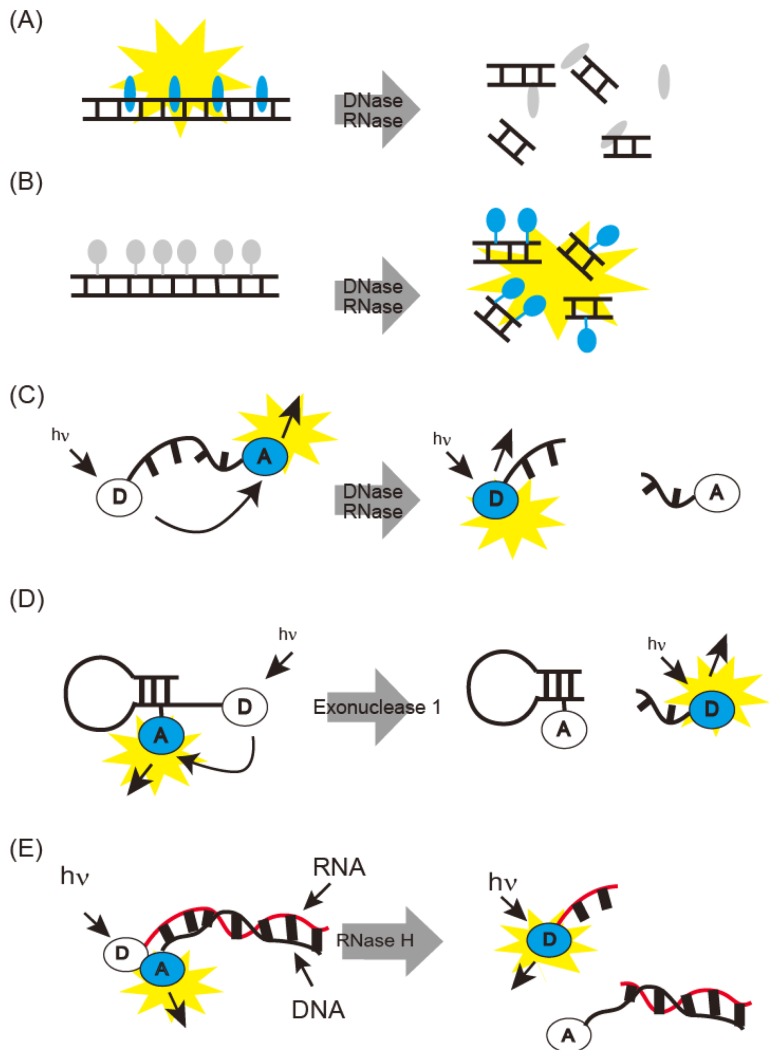
Fluorometric nuclease detection methods: (**A**) fluorescence intensity of non-covalent DNA-binding ligand; (**B**) recovery of self-quenched covalent fluorescent dye-DNA conjugate; (**C**) dequenching of FRET probes; (**D**) dequenching of hairpin-type FRET probes partially modified by phosphorothioate; and (**E**) de-hybridization of duplexes carrying FRET dyes after digestion with DNase, RNase, Exonuclease 1, or RNase H.

Fluorometric nuclease detecting methods are summarized in Figure 1. DNase I generates short DNA fragments by cleavage. Thus, DNase I can be detected as DNA fragmentation on gel electrophoresis. DNase I activity can also be monitored spectrophotometrically, due to the hyperchromic effect of the absorption region based on nucleotide bases [38]. PicoGreen fluoresces upon binding to double-stranded DNA and is used for fluorometric monitoring of short DNA fragments generated by DNase I (Figure 1A) [13]. Single radial enzyme diffusion (SRED) has also been developed for detection of DNase activity [8]. SRED is performed in agarose gel containing DNA and ethidium bromide (EtBr). Samples are spotted on the agarose gel; shortened EtBr-stained DNA fragments diffuse in the gel with DNase activity, and emit light. DNase I activity is estimated from the light-emitting area. Mammalian DNase I was classified by SRED into three types: pancreas, parotid, and their mixture [14].

DNase I detection sensitivity was improved by using a fluorescent dye-DNA covalent conjugate (Figure 1B) [39]. Fluorescence of this covalent conjugate was quenched and its fluorescent was recovered by degradation or digestion with DNase I. This method can detect picogram amounts of DNase I.

DNase I antibodies have been developed and used for enzyme-linked immunosorbent assay detection [40]. This is a convenient and sensitive method for measuring DNase content, though it does not necessarily reflect DNase activity.

Sophisticated methods have been developed for fluorescence resonance energy transfer (FRET) with oligonucleotides carrying a fluorophore and a quencher (Figure 1C) [15]. Fluorescence is quenched until DNase is added; then, the oligonucleotide is cleaved, releasing the chromophores; fluorescence intensity thus represents DNase activity. The oligonucleotides to be used are commercially available as DNaseAlert™ [15]. The detection limit of this probe is 5 × 10^−3^ U or 10 pg after 30 min at 37 °C and is thus rapid and highly sensitive. The FRET probe was also improved by the introduction of hairpin DNA sequences and partial displacement of phosphate with phosphorothioate (Figure 1D) [16]. These types of probes withstand the cellular environments, and sensitive detection of DNase I was realized with detection limit of 40 U/L. This probe was modified for Exonuclease I monitoring and provides intracellular fluorescence imaging. Simultaneous fluorescence imaging of DNase I and Endonuclease I activities was achieved by using two fluorophores (Figure 1).

Body fluids contain interferents that disrupt fluorescence detection. To remove them, DNase I-cleaved oligonucleotide fragments are purified before fluorescence detection [17]. Alternatively, DNase I is detected by using biotin- and fluorescein-modified PCR products immobilized on streptavidin-coated plates [18].

**Figure 2.**
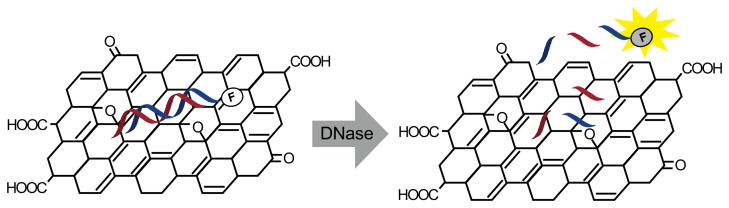
DNase I detection based on dequenching of fluorescence dye-labeled DNA duplexes on a GO surface.

Real-time fluorescence detection has been developed with graphene oxide (GO) and fluorescence-modified oligonucleotides (Figure 2) [19]. Dye fluorescence is quenched by adsorption on GO and is restored by desorption. When fluorescence-modified double-stranded oligonucleotides were mixed with GO, the oligonucleotide was spontaneously adsorbed and its fluorescence was quenched. DNase I digestion released the labeled oligonucleotides to fluoresce. The detection limit of this method is 1.75 U/mL in real time.

### 3.2. Electrochemical DNase Detection

**Figure 3.**
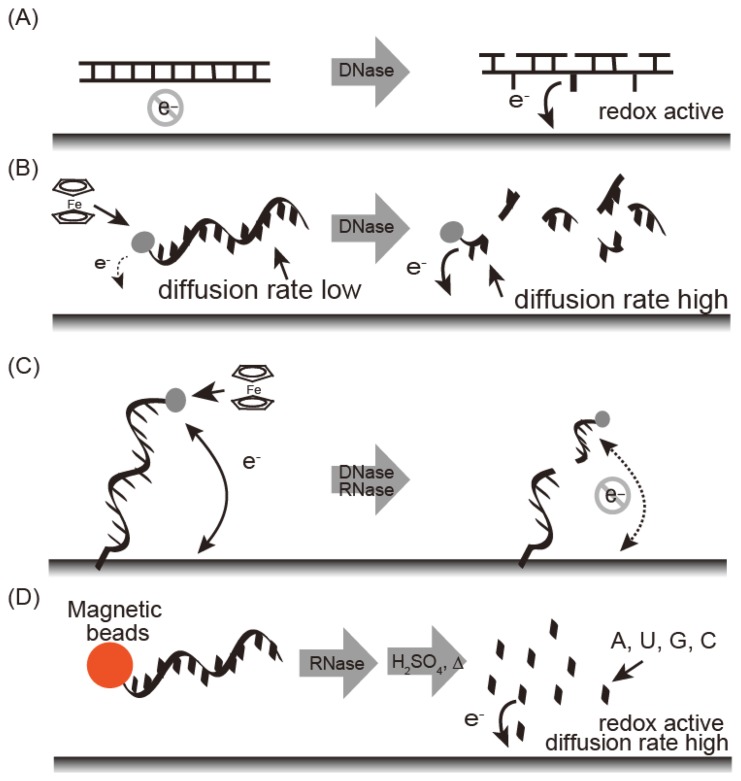
Electrochemical nuclease detection based on (**A**) direct redox of nucleobases exposed by nuclease digestion; (**B**) diffusion of ferrocene after fragmentation by nuclease; (**C**) loss of the ferrocene moiety from the electrode by digestion of the nuclease substrate; and (**D**) direct detection of nucleobases after nuclease digestion and hydrolysis.

Electrochemical nuclease detection methods are summarized in Figure 3. These methods are fast, easy to run, and require only compact instrumentation, thus allowing on-site diagnostics with a powerful detection tool. Electrochemical techniques are not affected by sample impurities. Nucleic bases undergo an electrochemical redox reaction and the reduction peak at −1.4 V as a reference for potential of standard calomel electrode (SCE) (*vs*. SCE) was observed for damaged DNA carrying exposed nucleic bases under adsorptive transfer stripping alternating voltammetry (AdTS AC voltammetry) [20,21]. Fojta and co-workers detected DNAse I by immobilizing plasmid DNA on an electrode (Figure 3A) [41]. Electrochemical nuclease detection with ferrocene oligonucleotide has been reported in homogenous solution (Figure 3B) [22]. Ferrocene is an electrochemically active signaling molecule that was immobilized with oligonucleotide to produce FcODN. In general, the observed current depends on the diffusion rate of the electrochemically active material; a larger current intensity is obtained with a smaller diffusion constant. Nuclease treatment with FcODN increased current intensity based on the ferrocene moiety with decreasing molecular weight as digestion progressed. Under differential pulse voltammetry, S1 nuclease, which is an endonuclease that degrades single-stranded regions of duplex DNA, was detected as an increasing peak current at 0.14 V *vs.* Ag/AgCl with the detection limit of 0.25 U/μL [23].

**Figure 4.**
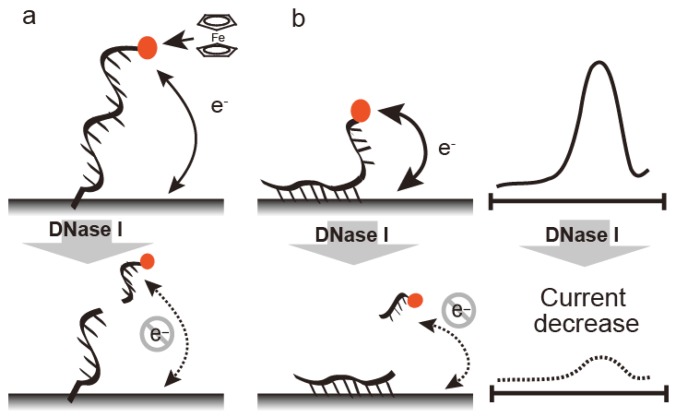
Nuclease detection by a ferrocene-modified oligonucleotide immobilized to an electrode through an Au-S linkage (**a**) and reaction of a cytosine oligonucleotide with activated ester-immobilized electrode (**b**). Current intensities at both electrodes decrease upon digestion with DNase I.

DNase can be detected by FcODN-immobilized electrodes, which provide real-time monitoring (Figure 3C). FcODN is immobilized on the electrode by Au-S linkages [23] (Figure 4A) or by reaction of cytosine moieties with amino moieties on the electrode surface (Figure 4B) [24]. DNase I addition causes cleavage of the ferrocene-oligonucleotide fragment and release from the electrode. Thus, the decrease in ferrocene causes a drop in the current, thus reflecting DNase I activity. DNase I was detected by a decrease in peak current at 0.18 V *vs.* Ag/AgCl under Osteryoung square wave voltammetry with a detection limit of 1 × 10^−4^ U/μL with an Au-S electrode or 1 × 10^−5^ U/μL with a cytosine-amino moiety linkage. These differences are derived from the orientation of the oligonucleotide on the electrode. Enzyme reactivity on a substrate-immobilized surface is depends on the orientation, mobility, and density of substrate on the electrode. DNase activity in blood may serve as a marker for breast cancer, oral cancer, or acute myocardial infarction. It is measured using an FcODN-immobilized electrode in the absence or presence of EDTA [23]. The presence of DNase I is indicated by a decrease in current in the presence of EDTA, which inactivates the enzyme. The accuracy of fluorometric and optical DNase I detection is limited by the turbidity of the blood sample; in contrast, the electrochemical technique is unaffected by sample turbidity.

**Figure 5.**
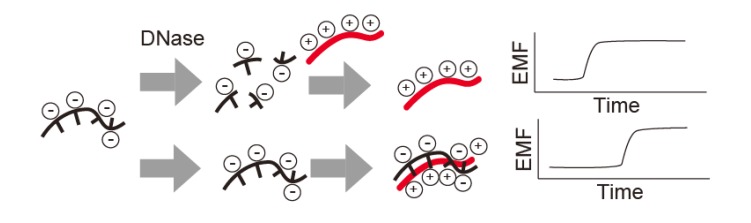
Nuclease detection with a polycation-sensitive membrane electrode based on the electromotive force (EMF) difference in the complexed polycationic polymer and DNA before and after digestion with DNase I.

DNase I can be detected by complexing DNA with a polycationic polymer (Figure 5). DNA is a polyanionic polymer that complexes with polycationic polymers, thus neutralizing its charge; however, digestion with DNase I destabilizes the polymer complex and misbalances the charge, generating an electromotive force that can be detected electrochemically. In one example of this technique, DNase I and S1 nuclease were detected at 4.5 × 10^−4^ U/μL and 3.2 × 10^−4^ U/μL, respectively, with protamine as a polycationic polymer and a polycation-sensitive membrane electrode [25].

Fluorometric S1 nuclease detection was also achieved by detecting fluorescent copper nanoparticles formed in the presence of a double-stranded DNA template [26]. S1 nuclease was detected from a decrease in the fluorescence at 560 nm with a detection limit of 0.3 U/mL. Both methods can detect DNase I or S1 nuclease with high sensitivity in homogeneous solution.

**Figure 6.**
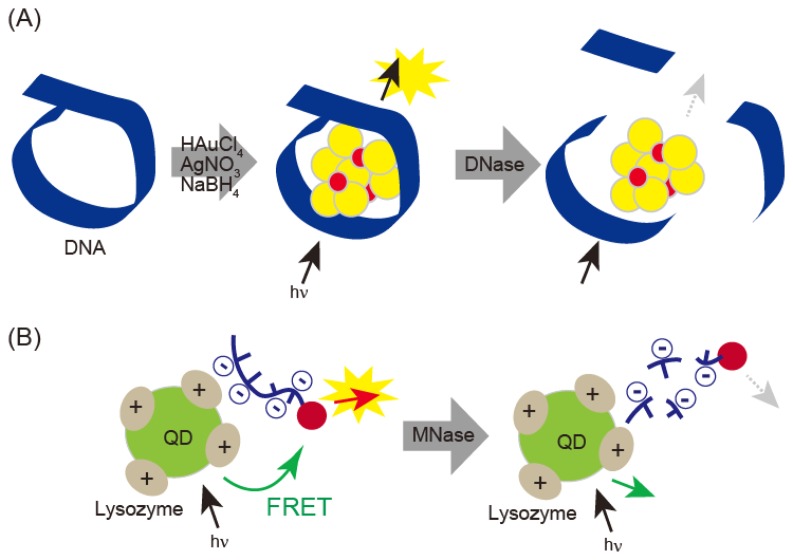
(**A**) DNase I detection of aggregated fluorescent DNA-covered nanoparticles after digestion with DNase I; (**B**) DNase I detection by electrostatic interaction-based FRET between positively charged QDs and negatively charged dye-labeled single-stranded DNA.

DNase I can be detected with fluorescent DNA-template gold/silver nanoclusters (DNA-Au/Ag NCs) (Figure 6A) [42]. When gold/silver nanoclusters were prepared in the presence of DNA template, fluorescence emission varied with size, which was influenced by the length of the DNA. When DNase I was treated with gold/silver nanoclusters, DNA was digested and the nanoclusters formed aggregates. DNase I was detected in saliva and serum samples and showed a linear correlation from 13 ng/mL to 60 μg/mL with a detection limit of 3 ng/mL. Quantitative detection of micrococcal nuclease (MNase) has been performed by electrostatic interaction-based fluorescence resonance energy transfer (FRET) between positively charged QDs and negatively charged dye-labeled single-stranded DNA (Figure 6B) [27]. This technique detects MNase concentrations over a range of 8 × 10^−3^ to 9.0 × 10^−2^ unit·mL^−1^, with a limit of detection of 1.6 × 10^−3^ unit·mL^−1^.

## 4. RNase Detection

### 4.1. Fluorometric RNase Detection

**Figure 7.**
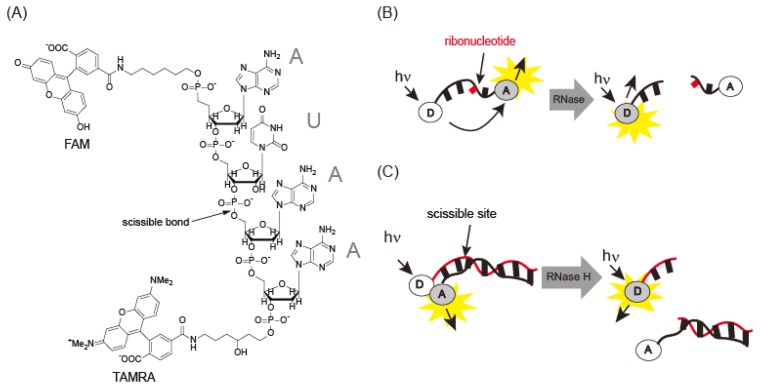
(**A**) Chemical structure of the FRET probe for RNase A detection; (**B**) RNase A and (**C**) RNase H detection with FRET probes.

RNase A is an endonuclease analogous to DNase I. Uridine 3′-(p-nitrophenylphosphate) or uridine 3′-nitorophenylphosphate-(5-bromo-4-chloroindol-3-yl)-phosphate has been used for spectrophotometric detection of RNase [28]. However, RNase A has low *k*_cat_/*K*_m_ values for these substrates. Rains and co-workers [29] identified AUAA or ACAA as a good substrate sequence for RNase A. A FRET probe labeled with two chromophores (6-FAM-dArUdAdA-6-TAMRA) was synthesized to detect RNase A activity (Figure 7A,B). FAM fluorescence was quenched by TAMRA and increased 180-fold after cleavage of the ribouridine (rU) site by RNase. This probe exhibited a *k*_cat_/*K*_m_ of 3.6 × 10^7^M^−1^·s^−1^, 10-fold greater than previous reports. Angiogenin, an RNase A associated with cancer, exhibited a *k*_cat_/*K*_m_ of 3.3 × 10^7^M^−1^·s^−1^, similar to the value for RNase A. This probe was used in an inhibition assay with 3′-UMP and 5′-ADP [29]. Digestion of 0.6 μM fluorescence probe with 0.57 pM RNase A was performed in the presence of 3′-UMP and 5′-ADP, yielding inhibition constants, *K*_i_s, of 60 μM and 8.4 μM for 3′-UMP and 5′-ADP, respectively. Thus, the probe can be used to detect RNase activity and as a screening reagent for angiogenin. Another type of fluorescence probe is composed of a duplex between a fluorescein-modified RNA strand and a dabcyl-DNA strand [30]. Although fluorescence is quenched by dabcyl through FRET, it is recovered after treatment with RNase H, which cleaves the RNA strand in the DNA-RNA hybrid (Figure 7C). FRET probes containing ribonucleotides make it possible to monitor RNase H, even in the cell.

### 4.2. Electrochemical RNase Detection

**Figure 8.**
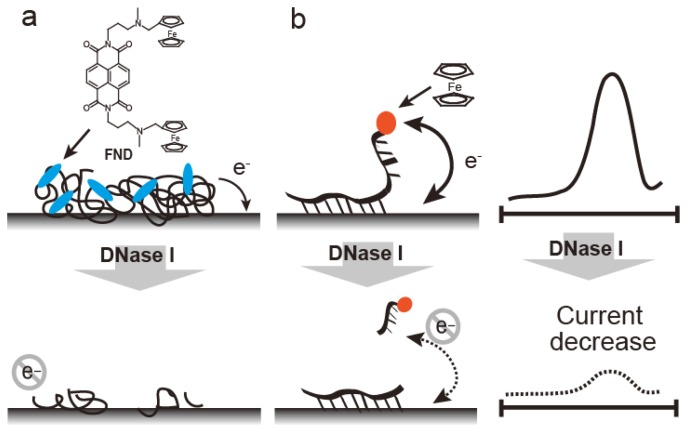
RNase detection with mRNA immobilized on a glassy carbon electrode (**a**) or ferrocene oligonucleotide with a ribonucleoside unit immobilized via continuous cytosine and the activated ester electrode (**b**). Current intensities decrease with RNase A activity.

Electrochemical methods, such as those involving ferrocenyl naphthalene diimide (FND) coupled to an mRNA-bound electrode, are used to detect RNase (Figure 8) [43]. FND has been developed as an electrochemical DNA duplex indicator [31,44], but FND also bound to mRNA with relatively low affinity, which could be due to the higher order structures containing double stranded regions within the mRNA. FND can be absorbed into mRNA-immobilized electrode, which allows FND concentration on the mRNA-immobilized electrode (Figure 8a). When the electrode was treated with RNase A, the amount of mRNA on the electrode decreased. RNase A activity is represented by the decreasing current with a linear range of 0.2–10 ng/mL and a detection limit of 0.2 pg. RNase A detection was performed by electrode-immobilized FcODN containing dArCdAdA and continuous cytosines (Cs) (Figure 8b) [32]. This FcODN was immobilized on the electrode through reaction of its continuous Cs with activated ester moieties coating the electrode surface. When the rC of FcODN on the electrode was cleaved by RNase A, FcODN fragments containing ferrocene were released, reducing the current, which is detected as RNase A activity. This electrode provides quantitative detection of RNase A from 1 × 10^−11^ g/mL to 1 × 10^−6^ g/mL by Osteryoung square wave voltammetry. Guanidine thiocyanate and EDTA inhibit the activity of RNaseA and DNase I, respectively; the specificity of the FcODN-immobilized electrode for these enzymes is derived from these properties.

**Figure 9.**
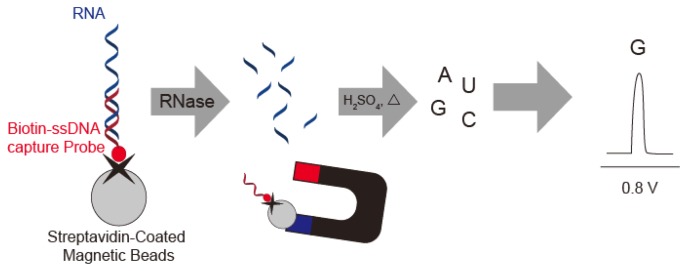
RNase digestion and dipurinization is followed by stripping chronopotentiometric measurements with a graphite pencil electrode to produce a guanine redox peak.

Wang's group described a sensitive method for detection of RNase A by using substrate oligonucleotides immobilized on magnetic beads. After digestion with RNase A and acid dipurinization, the guanine bases were separated from the magnetic beads and detected by stripping chronopotentiometric measurements with a graphite pencil electrode (Figure 9). RNase A activity is estimated by the current peak at 0.8 V *vs.* Ag/AgCl from the guanine base. The authors were thus able to detect 4 × 10^−12^ g/mL RNase A using the lowest potential based on a guanine base [45].

## 5. Conclusions

DNase and RNase can be detected by fluorometric and electrochemical techniques with several kinds of synthetic oligonucleotide substrates. Drug discovery will be facilitated by high-throughput screening of DNase and RNase drugs with a fluorometric technique in a microtiter format; in addition, electrochemical techniques are useful for detecting enzymes associated with disease. These techniques are especially good at producing data even in the presence of interfering substances. Table 2 shows the latest reports of DNase and RNase detection; nanoparticles were used in No. 2, 5, and 6 in Table 2. Finally, the development of improved nuclease probes will help clarify the function of DNase and RNase and the mechanism of their involvement in disease.

## Figures and Tables

**Table 1.** Analytical performance of DNase and RNase detection platforms.

**No.**	**Sensing Platform**	**Target**	**Detection Limit**	**Linear Range**	**[Reference]**
**1**	Single radial enzyme diffusion method	DNase I	15.6 ng/mL	15.6–500 mg/mL	[8]
**2**	Fluorometric detection with dsDNA coupled with picogreen	DNase I	5 pg	1–100 pg	[14]
**3**	DNaseAlert^®^	DNase I	0.005 unit	0.005–0.5 unit	[15]
**4**	FRET dsDNA probe	DNase I	40 unit/L	40–500 unit/L	[16]
		Exonuclease	2.0 unit/L	2.0–400 unit/L	
**5**	Fe_3_O_4_^@^MIP nanoparticle	DNase I	-	-	[17]
**6**	Immunochemical detection using 794 bp PCR product labeled fluorescein and biotin	DNase II	0.05 unit/mL	0.025–4 unit/mL	[18]
**7**	Graphene-based fluorescence assay	DNase I	1.75 unit/mL	1.75–70 unit/mL	[19]
**8**	Electrochemical detection based single stranded break in homogeneous solution	DNase I	0.6 ng/mL	0.6–1000 ng/mL	[20]
**9**	Electrochemical detection based single stranded break with DNA modified electrode	DNase I	0.2 ng/mL	-	[21]
**10**	Electrochemical detection using FcODN in homogeneous solution	S1 nuclease	0.25 unit/μL	-	[22]
**11**	Electrochemical detection using FcODN modified electrode (Au-S linkage)	DNase I	10^−4^ unit/μL	10^−4^–10^−2^ unit/μL	[23]
**12**	Electrochemical detection using FcODN modified electrode (cytosine oligonucleotide)	DNase I	10^−5^ unit/μL	10^−5^–10^−3^ unit/μL	[24]
**13**	Potentiometric sensing using polycation sensitive membrane electrode	S1 nuclease DNase I	0.32 unit/mL 0.45 unit/mL	- 1–10 unit/mL	[25]
**14**	ds DNA template formation Cu nanoparticle	S1 nuclease	0.3 unit/mL	1–50 unit/mL	[26]
**15**	DNA template gold/silver nanocluster	DNase II	3 ng/mL	0.013–60 μg/mL	[27]
**16**	positively charged QDs-based FRET probe	MNase	1.6 unit/L	8–90 unit/L	[28]
**17**	RNaseAlert^®^	RNase A	0.25 pg	0.25–2.0 pg	[15]
**18**	FRET dsDNA-RNA probe	RNase H	28 pM	30–120 pM	[29]
**19**	Electrochemical detection using mRNA modified electrode	RNase A	0.2 pg	0.2–10 pg	[30]
**20**	Electrochemical detection using FcODN modified electrode (cytosine oligonucleotide)	RNase A	10 pg/mL	1 pg/mL–1 μg/mL	[31]
**21**	RNA modified magnetic nanoparticle	RNase A	10^−8^ unit (4 pg/mL)	9.2 × 10^−9^–9.2 × 10^−4^ unit	[32]

**Table 2.** Analytical performance in platforms of DNase and RNase detection (2012–2013).

**No.**	**Sensing Platform**	**Target**	**Detection Limit**	**Linear Range**	**[Reference] (year)**
**1**	FRET dsDNA probe	DNase I	40 unit/L	40–500 unit/L	[16]
		Exonuclease	2.0 unit/L	2.0–400 unit/L	(2013)
**2**	Fe_3_O_4_@MIP nanoparticle	DNase I	-	-	[17]
					(2013)
**3**	Graphene-based fluorescence assay	DNase I	1.75 unit/mL	1.75–70 unit/mL	[19]
					(2012)
**4**	Potentiometric sensing using polycation sensitive membrane electrode	S1 nuclease	0.32 unit/mL	−	[25]
		DNase I	0.45 unit/mL	1–10 unit/mL	(2013)
**5**	ds DNA template formation Cu nanoparticle	S1 nuclease	0.3 unit/mL	1–50 unit/mL	[26]
					(2013)
**6**	Positively charged QDs-based FRET probe	MNase	1.6 unit/L	8–90 unit/L	[27]
					(2013)
**7**	Electrochemical detection using FcODN modified electrode (cytosine oligonucleotide)	RNase A	10 pg/mL	1 pg/mL–1 μg/mL	[31]
					(2013)
